# Descending Vasa Recta Endothelial Membrane Potential Response Requires Pericyte Communication

**DOI:** 10.1371/journal.pone.0154948

**Published:** 2016-05-12

**Authors:** Zhong Zhang, Kristie Payne, Thomas L. Pallone

**Affiliations:** Division of Nephrology, Department of Medicine, University of Maryland School of Medicine, Baltimore, Maryland 21201, United States of America; Charité Universitätsmedizin Berlin, GERMANY

## Abstract

Using dual-cell electrophysiological recording, we examined the routes for equilibration of membrane potential between the pericytes and endothelia that comprise the descending vasa recta (DVR) wall. We measured equilibration between pericytes in intact vessels, between pericytes and endothelium in intact vessels and between pericytes physically separated from the endothelium. Dual pericyte recording on the abluminal surface of DVR showed that both resting potential and subsequent time-dependent voltage fluctuations after vasoconstrictor stimulation remained closely equilibrated, regardless of the agonist employed (angiotensin II, vasopressin or endothelin 1). When pericytes where removed from the vessel wall but retained physical contact with one another, membrane potential responses were also highly coordinated. In contrast, responses of pericytes varied independently when they were isolated from both the endothelium and from contact with one another. When pericytes and endothelium were in contact, their resting potentials were similar and their temporal responses to stimulation were highly coordinated. After completely isolating pericytes from the endothelium, their mean resting potentials became discordant. Finally, complete endothelial isolation eliminated all membrane potential responses to angiotensin II. We conclude that cell-to-cell transmission through the endothelium is not needed for pericytes to equilibrate their membrane potentials. AngII dependent responses of DVR endothelia may originate from gap junction coupling to pericytes rather than via receptor dependent signaling in the endothelium, per se.

## Introduction

Descending vasa recta (DVR) are arteriolar microvessels that supply blood flow to the renal medulla. Their wall is comprised of a continuous endothelium surrounded by smooth muscle-like pericytes [1–3] whose contractile activity involves activation of voltage gated calcium entry pathways [4, 5]. In turn, the membrane potential that governs voltage gated calcium channel (VGCC) activity is largely governed by the balanced activities of K^+^ and Cl^-^ channel conductance. Application of a vasoconstrictor, such as angiotensin II (AngII) activates calcium dependent chloride channels (CaCC) and leads to delayed suppression of K^+^ channels leading to depolarization [6, 7]. An important feature of the DVR wall, and microvessels of other organs in general, is that mural cells (smooth muscle, endothelia) can modify one another’s membrane potential by electrical conduction across gap junctions [8, 9]. It is generally believed that such intercellular communication in small microvessels can extend the range of stimuli at one location so to reach distant points along the vessel axis. In the renal medulla, for example, this favors the hypothesis that mediators released in response to outer medullary hypoxia might transmit their actions through the DVR syncytium to dilate upstream, parent juxtamedullary efferent arterioles.

Experimental modification of syncytial communication between smooth muscle (e.g., pericytes) and endothelium is difficult because chemical gap junction blockade can have nonspecific effects on channel activity and other membrane proteins [10]. Similarly, genetic disruption of constituent connexin subunits that comprise gap junctions may alter expression of other connexin isoforms or modify the heterologous combinations that form assembled connexon proteins [9]. In this effort we used an alternative approach, physical disruption of contact between cells, to test for presence of communicative pathways. We removed abluminal pericytes and performing dual-cell homologous or heterologous recording from pericytes and endothelia to assess the persistence of membrane potential equilibration, both at rest and during response to contractile agonists. In some experiments, pericytes were fully separated from the endothelium to test whether such disruption modifies or eliminates responses of either cell type to AngII. We found that all pericytes on an isolated DVR segment maintain equal resting potentials and that such coupling persists during depolarization from AngII, arginine vasopressin (AVP), or endothelin 1 (ET1). No constrictor modified capacity for pericyte-to-pericyte coordination. Pericytes fully separated from one another depolarized but could not coordinate their responses. Finally, after complete pericyte removal, depolarizing responses in the isolated endothelium was eliminated. These results show that pericyte-to-endothelial electrical conduction is not needed to coordinate pericyte response; however pericyte contact is needed to achieve endothelial response.

## Materials and Methods

### Isolation of DVR

This study was carried out in strict accordance with the recommendations in the Guide for the Care and Use of Laboratory Animals of the National Institutes of Health. The protocol was approved by the Committee on the Ethics of Animal Experiments of the University of Maryland (IACUC Protocol number: 1113009). All surgery was performed under ketamine / xylazine anesthesia, and all efforts were made to minimize suffering. Sprague Dawley rats (120–200 g; Harlan) were anesthetized by an intraperitoneal inj*e*ction of ketamine / xylazine (80 mg/kg / 10 mg/kg), following which kidneys were harvested and sectioned as previously described [7, 11]. Tissue slices were stored at 4°C in physiological saline solution (PSS, in mM) NaCl 145, KCl 5, MgCl_2_ 1, CaCl_2_ 1, N-2-hydroxyethylpiperazine-N'-2-ethanesulfonic acid (HEPES) 10, osmolality ~356 mOsm/Kg H_2_O, glucose 10, pH 7.4 at room temperature). To permit formation of gigaseals during patch clamp, tissue wedges comprised of cortex, outer and inner medulla were digested at 37°C for ~22 minutes in a collagenase 1A (0.5 mg/ml), protease XIV (0.4 mg/ml) and bovine serum albumin (1.0 mg/ml) in calcium free PSS. After digestion, the tissue was stored in calcium replete PSS at ~4°C. DVR segments were isolated from inner stripe vascular bundles by hand dissection and transferred to a chamber on an inverted microscope for patch clamp [7]. Access to endothelia was achieved by complete or partial pericyte removal from the abluminal surface. As previously described and illustrated, pericytes where sheared off the wall of collagenase treated vessels when they were drawn into, and then ejected from, a micropipette whose orifice had been heat polished to ~6 microns [12].

### Whole cell patch clamp recording

Patch clamp electrodes were constructed from borosilicate capillary tubes (PG52151-4, external diameter 1.5 mm, internal diameter 1.0 mm; World Precision Instruments, Sarasota, FL), with a two-stage vertical pipette puller (Narshige PP-830) followed by heat polishing of their tips. Whole-cell electrical access was achieved using nystatin (100 microg/ml) as the pore forming agent [6, 7]. The electrode buffer was (in mmol/L): KAspartate 120, KCl 20, NaCl 10, HEPES 10, osmolality ~320 mOsm/Kg H_2_O, pH 7.2. The extracellular buffer was PSS. The bath was introduced by gravity into a custom constructed chamber and drawn away by suction. The geometry of the chamber has been previously described [7]. Briefly, it has a narrow channel where the bath electrode Ag/AgCl wire is placed. The preparation is immobilized just downstream of the wire and buffer entry point. Downstream of the narrow channel the chamber enlarges to a wide portion where flowing bath is removed by suction. This configuration provides for rapid exchange of reagents in the vicinity of the preparation with minimization of electrical noise. Dual cell recordings were performed at room temperature with a Multiclamp 700B amplifier as previously described [13]. Quality of gigaseals was verified using Clampex 10 and membrane potential was recorded with zero current clamp protocols [7]. In some voltage clamp experiments we imposed square wave membrane potential deviations on a pericyte from a holding level of -80 mV to -40 mV while recording membrane potential of distant endothelial cells by zero current clamp at 10 Hz. Those protocols were executed with Clampex and the recordings digitized using a Digidata 16 bit analog-to-digital converter (Molecular Devices). Results were corrected for junction potentials [7].

### Reagents

Nystatin, angiotensin II, vasopressin, endothelin, heptanol 18BGRA, collagenase 1A, protease XIV and other chemicals were from Sigma (St Louis, MO). Reagents were thawed and diluted on the day of the experiment and excess discarded daily.

### Statistics

Data in the text and figures are reported as mean ± SE. For all protocols, one DVR was analyzed per rat for inclusion in the summarized results. Experiments in which gigaseals failed before or during data acquisition were discarded. Correlations were analyzed by linear regression. Sequential measurements in individual DVR were analyzed by repeated measures ANOVA using Holm-Sidak post hoc multiple comparison testing with SigmaStat 3.11 (Systat Software, Inc., Point Richmond, CA).

## Results

### Dual pericyte membrane potential recording, intact DVR

**Fig 1:**
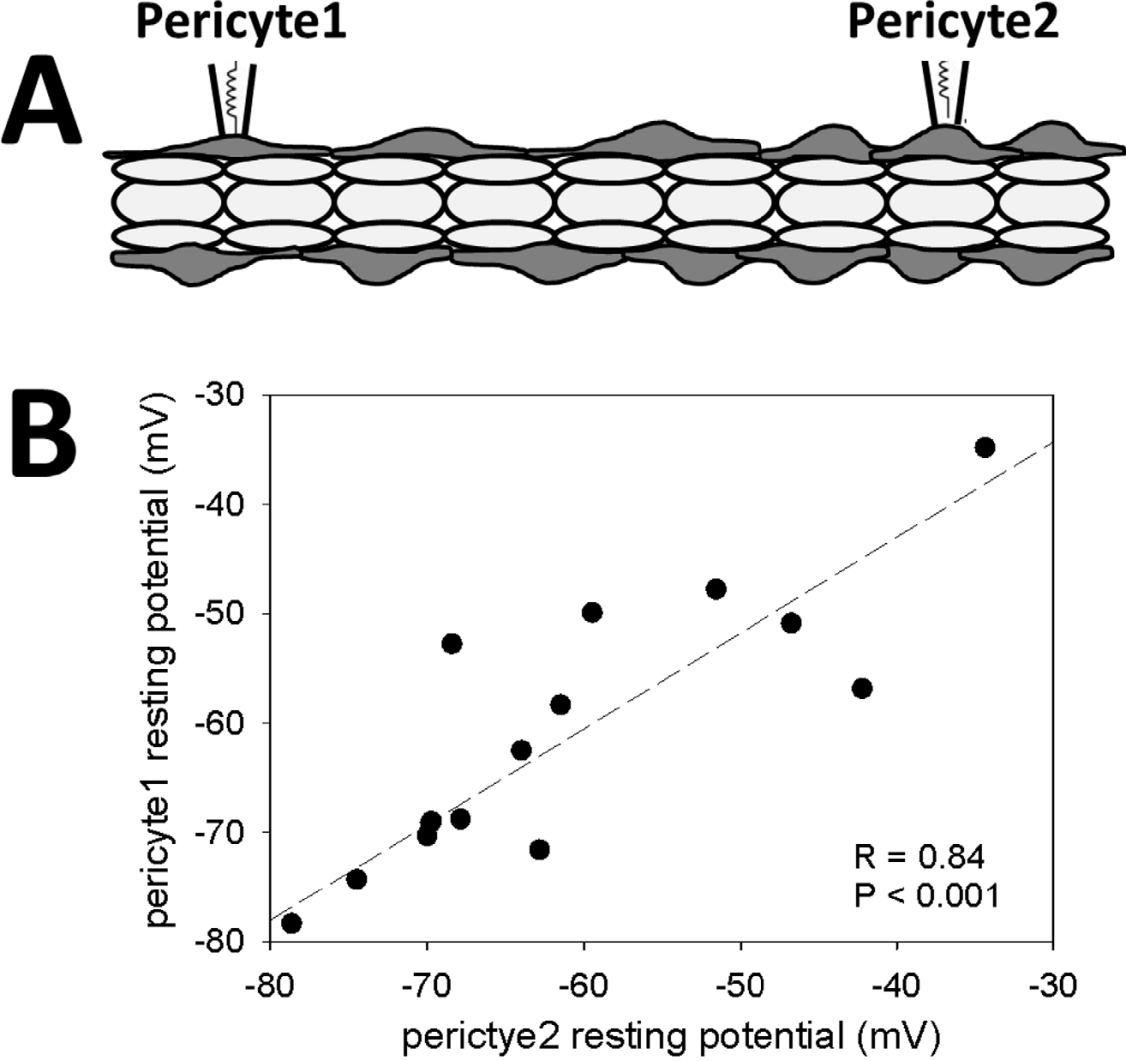
Dual pericyte patch clamp configuration and resting potentials. **A.** Dual recordings of membrane potential were performed on pericytes (dark gray) of intact DVR with endothelium (light gray) present. **B.** Each data point shows the initial resting potentials recorded from two pericytes of the same DVR (N = 15). The dashed line shows the best fit linear regression (R = 0.84, P < 0.001).

In a first series, we tested the extent to which pericytes of a single DVR share membrane potential, both at rest and during agonist induced depolarizations. Three agents known to constrict DVR were studied, AngII (10 nM), AVP (100 nM) and ET1 (1 nM). We chose the respective concentrations to match those that generated maximal vasoconstriction of microperfused DVR in past studies [14–16]. Dual pericyte recordings were performed in DVR from 5 rats per constrictor (1 DVR per rat, N = 15 rats total). No vessel was exposed to more than one agent. The correlation between resting membrane potentials is shown in **Fig 1** where the dashed line is the best-fit linear regression (F = 31.8, DF = 14, R = 0.84, P < 0.001). Close approximation of resting potential of pericytes along the intact DVR wall was consistently observed (pericyte1 vs pericyte2, mean ± SEM: -61 ± 2.7 mV vs -61 ± 2.6 mV, N = 15).

**Fig 2:**
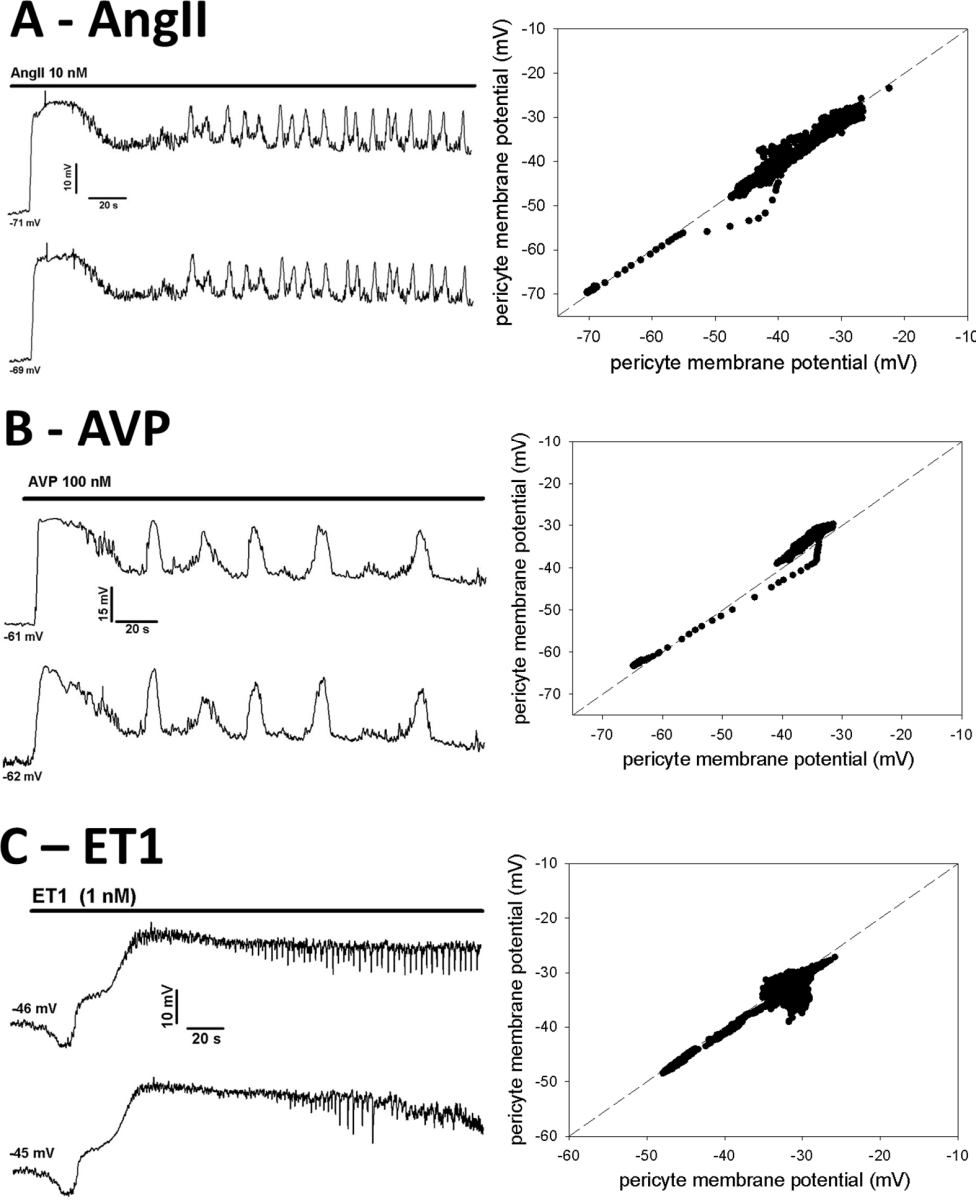
Simultaneous membrane potential recording in two pericytes during exposure to vasoconstrictors. **A. AngII:** Left panel shows an example of membrane potential responses of two pericytes on a DVR during exposure to AngII (10 nM, similar to n = 5). Resting potentials prior to AngII were -71 mV and -69 mV. **B. AVP:** Left panel shows an example of membrane potential responses of two pericytes during exposure to AVP (100 nM, similar to n = 5). Resting potentials prior to exposure were -61 mV and -62 mV. **C. ET1:** Left panel shows an example of membrane potential responses of two pericytes during exposure to Endothelin 1 (ET1, 1 nM, similar to n = 5). Resting potentials prior to exposure were -46 mV and -45 mV. For all constrictors, temporally similar variations of paired pericytes persisted throughout the recordings. Right panels show dashed lines of identity with individual data points of the adjacent records superimposed upon them.

Examples of time-dependent changes in pericyte membrane potential during exposure to vasoconstrictors are shown in **Fig 2**. Regardless of the agent used, AngII (**Fig 2A**), AVP (**Fig 2B**), or ET1 (**Fig 2C**), depolarization of two pericytes always occurred together in a similar pattern with membrane potentials retaining near-identity. Syncytial coordination was maintained and no agent interfered with gap junction coupling. We calculated the difference between pericyte membrane potentials at each time point during the responses and averaged the results to quantify the degree of equilibration. Those time-averaged differences were 0.29 ± 0.34 mV, 1.5 ± 1.20 mV, and 0.29 ± 0.34 mV (mean ± SEM) for AngII, AVP and ET1, respectively. The differences were not significantly different from zero (within two standard errors). There were no significant differences between time averaged pericyte membrane potentials for any vasoconstrictor (paired t-test) nor was there a difference between the magnitude of depolarization achieved by AngII, AVP or ET1 (ANOVA). Summaries of the equilibrations are provided in **Table 1** where the correlation coefficients (R values), number of data points recorded and P values for the linear regressions have been summarized as mean ± SEM.

**Table 1:** Summary of linear regressions fit to dual pericyte membrane potential records during vasoconstrictor application.

	Configuration	Data points per record^a^	R value for linear regression^b^	n^c^	
**AngII (10 nM)**	Intact DVR	3750 ± 631	0.92 ± 0.06	5	NS ^d^
**AVP (100 nM)**	Intact DVR	2538 ± 498	0.94 ± 0.04	5	NS ^d^
**ET1 (1 nM)**	Intact DVR	2234 ± 526	0.94 ± 0.01	5	NS ^d^
**AngII (10 nM, P-P)**	Isolated pericyte	3007 ± 624	0.91 ± 0.04	8	
**AngII (10 nM, PxP)**	Isolated pericyte	2800 ± 314	0.35 ± 0.05	8	P < 0.01 vs P-P

a, the mean ± SE of data points recorded for each vasoconstrictor

b, the mean ± SE of the “R” correlation coefficients of the linear regressions

c, n = number of recorded pericyte pairs = number of vessels = number of rats

d, NS; R values not significantly different from other vasoconstrictors by ANOVA.

### Dual recordings from pericytes in isolation

**Fig 3:**
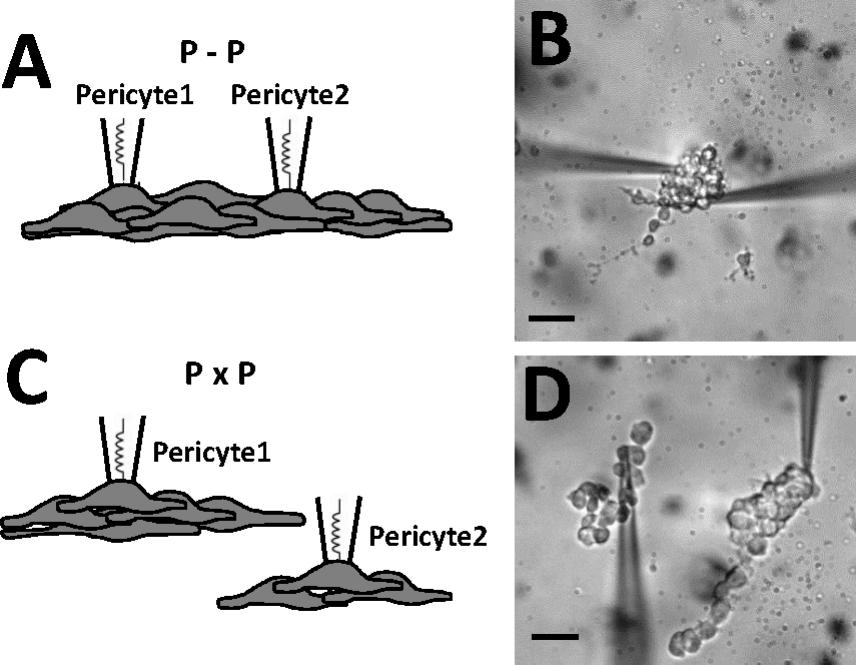
Simultaneous membrane potential recording from isolated pericytes; pericytes in contact. Schematic drawings and photomicrographs of dual recording from isolated pericytes in contact (**A, B,** designated P-P) on a coverslip or isolated without contact (**C, D,** designated PxP). The black bars = 10 microns.

**Fig 4:**
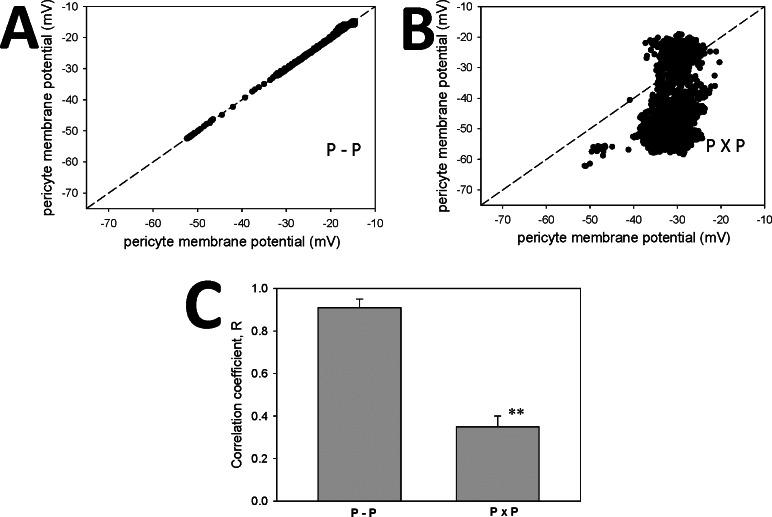
**A.** Single examples of AngII (10 nM) stimulated membrane potentials of paired pericytes isolated from endothelium, either in contact (**A**, P-P) or without contact (**B**, PxP). In each example the individual data points are superimposed upon the dashed line of identity. **C.** Summary of mean ± SEM of correlation coefficients for P-P and PxP configurations (n = 8 each; **, P < 0.01). Other summaries are provided in **Table 1**.

The close similarity of membrane potentials between pericytes (**Figs 1 and 2**) might be accounted for by conduction between pericytes, per se, or by conduction from pericyte to endothelium and back to nearby pericytes. To determine whether pericyte-to-pericyte conduction could account for the coordination, we removed pericytes from the DVR wall and placed them on coverslips. Two configurations were used, one in which accessed pericytes retained physical contact with one another (**Fig 3A and 3B,** abbreviated P-P), and another where spatial separation was imposed (**Fig 3C and 3D,** abbreviated PxP). Dual recordings during AngII (10 nM) stimulation derived from pericytes “in contact” (**Fig 4A, P-P**) yielded coordinated depolarizations like those in **Fig 2A**. In contrast, when cell-to-cell contact was disrupted (**Fig 4B, PxP**), pericyte depolarizations occurred but were not synchronized. Comparison of the mean ± SEM of correlation coefficients for n = 8 experiments in each configuration is shown in **Fig 4C** and summarized along with results from **Fig 2** in **Table 1.** Pericytes can share membrane potential and synchronize their responses in the absence of an endothelial layer.

### Dual pericyte, endothelial recordings

**Fig 5:**
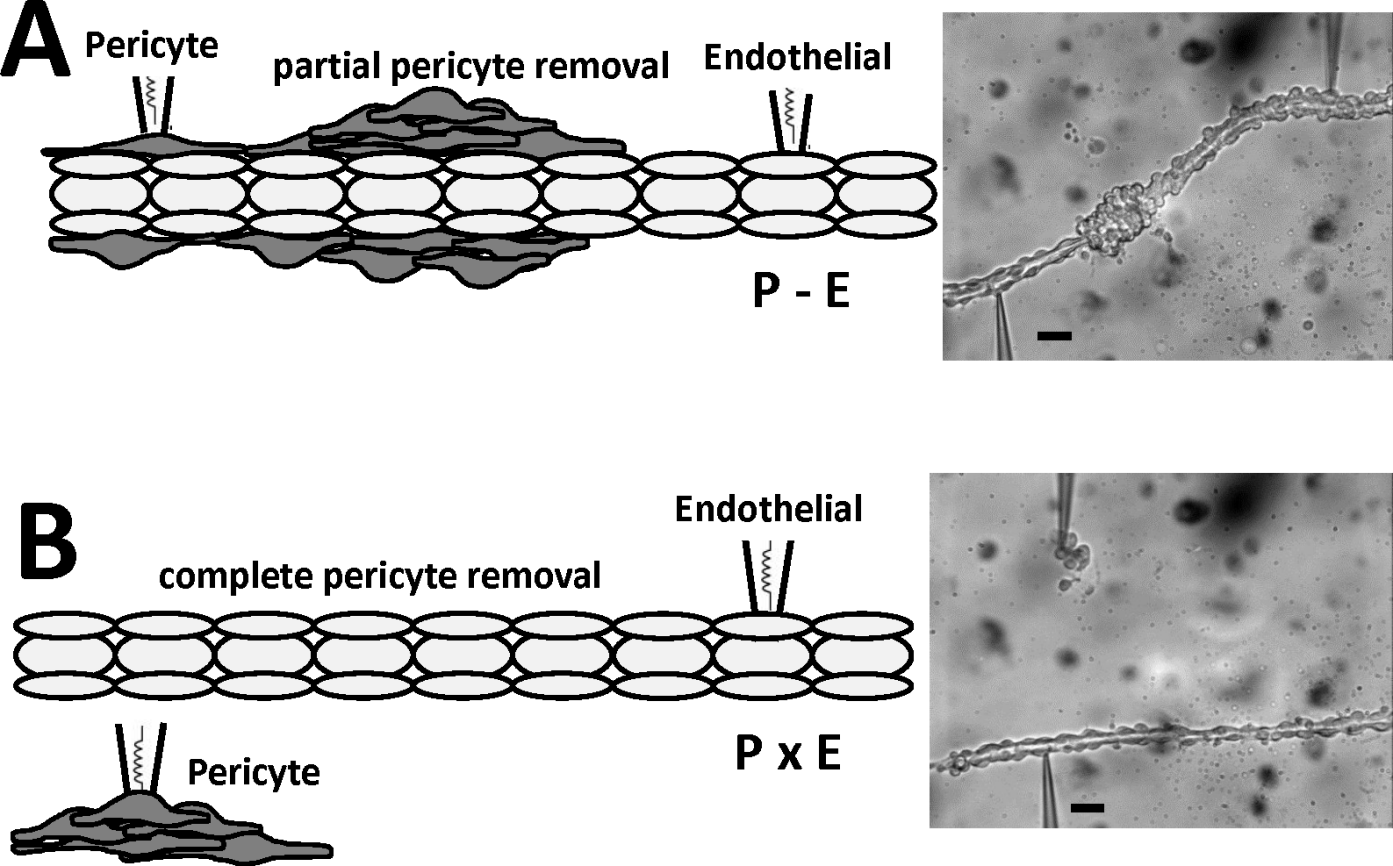
Configurations for simultaneous recording from DVR pericytes and endothelia. **A.** Left and right panels show schematic depiction and photomicrograph of a partially denuded DVR with simultaneous dual-cell patch clamp of a pericyte and an endothelial cell that retain contact (abbreviated P-E). **B.** Left and right panels show a schematic depiction and photomicrograph of a DVR, fully denuded of pericytes, with simultaneous dual-cell patch clamp of each cell type when they have no contact (abbreviated PxE). These configurations were used to test the importance of cell contact for AngII dependent membrane potential responses. The black bars = 10 microns.

**Fig 6:**
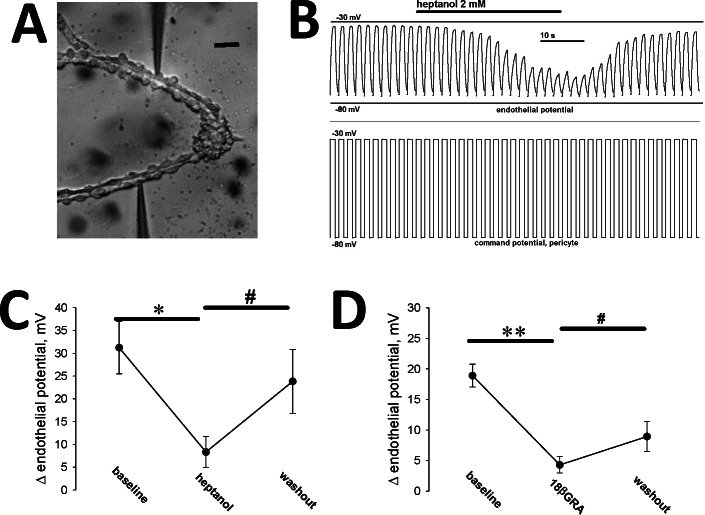
Dual recording from pericyte and endothelium during AngII exposure and gap junction blockade. **A.** Photomicrograph of the dual pericyte, endothelial preparation that yielded the recording in panel B. The black bar = 10 microns. **B.** Electrophysiological record; square wave depolarizations from -80 to -30 mV were imposed at regular intervals on a voltage clamped pericyte. The endothelial response during pericyte depolarization (top) and the pericyte command potential (bottom) are shown before, during and after application of heptanol (2 mM). **C.** Summary of experiments similar to that shown in panel B. The average membrane potential deviation of the endothelium is shown before, during and after washout of heptanol, which reversibly attenuated conduction (mean ± SEM, n = 6). Repeated measures ANOVA, DF = 17, F = 19.5, P < 0.001. By Holm-Sidak multiple comparison; * P < 0.05 baseline vs heptanol, # P < 0.05, heptanol vs washout. **D.** Summary of experiments similar to that shown in panel B, but with 18BGRA (20 microM) as the gap junction blocker (mean ± SEM, n = 8). Repeated measures ANOVA, DF = 23, F = 38.6, P < 0.001. By Holm-Sidak multiple comparison; ** P < 0.01 baseline vs 18BGRA, # P < 0.05 18BGRA vs washout.

We have previously shown that AngII stimulation depolarizes both the pericyte and endothelial layers of the DVR wall [17, 18]. To specifically test the separate ability of pericytes and endothelia to respond to AngII, we performed simultaneous dual-cell recording with configurations illustrated in **Fig 5**. Pericytes were either partially sheared from the vessel wall (**Fig 5A,** abbreviated P-E), so that the patched pericytes and endothelia remained physically associated, or pericytes were completely removed and deposited onto a coverslip adjacent to the denuded endothelium (**Fig 5B,** abbreviated PxE). In one series we used voltage clamp protocols to impose square wave membrane potential depolarizations on a pericyte while recording transmission to a distant endothelial cell. We then introduced gap junction blockade heptanol, (2 mM) or 18B-glycyrrhetinic acid (18BGRA, 20 microM) to interfere with cell-to-cell transmission. As illustrated in the example **Fig 6A and 6B**, the square-wave command potential imposed on the pericyte was reproduced distantly in the endothelium. The communicated endothelial response was reversibly attenuated, but not eliminated, by gap junction blockade with heptanol (**Fig 6B**). Averaged results for heptanol are shown in **Fig 6C** (n = 6). Averaged results for 18BGRA are shown in **Fig 6D** (n = 8).

**Fig 7:**
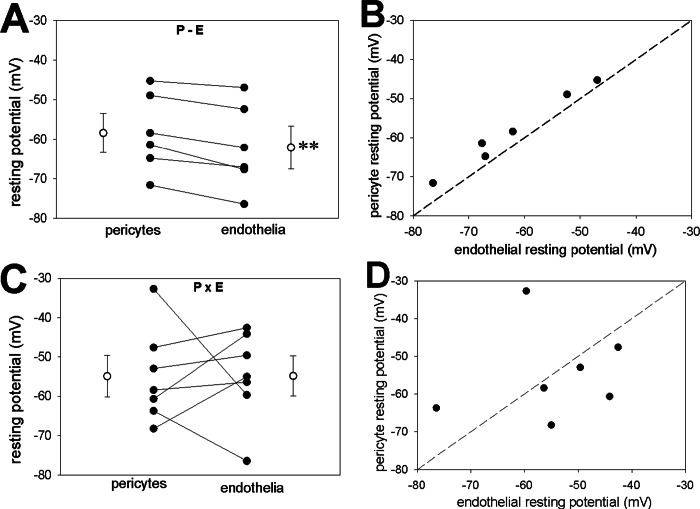
Dual recording from pericytes and endothelia during AngII exposure, cells associated or spatially separated. **A.** Resting potential of pericytes and endothelia that are associated on the DVR wall (as in Fig 5A). Endothelial cells were significantly hyperpolarized relative to the pericytes (paired t-test, n = 6, ** P < 0.01). **B.** Simultaneous endothelial and pericyte resting membrane potentials from panel A superimposed on the line of identity. By linear regression, values were highly correlated (DF = 5, F = 221, R = 0.99, P < 0.001). **C.** Resting potential of pericytes and endothelia dissociated from one another (as in Fig 5B). Resting potentials were not significantly different (n = 7). **D.** Simultaneous endothelial and pericyte resting membrane potentials from panel C superimposed on the line of identity. A significant correlation was not observed (DF = 6, F = 0.13, R = 0.16, P = 0.73).

**Fig 8:**
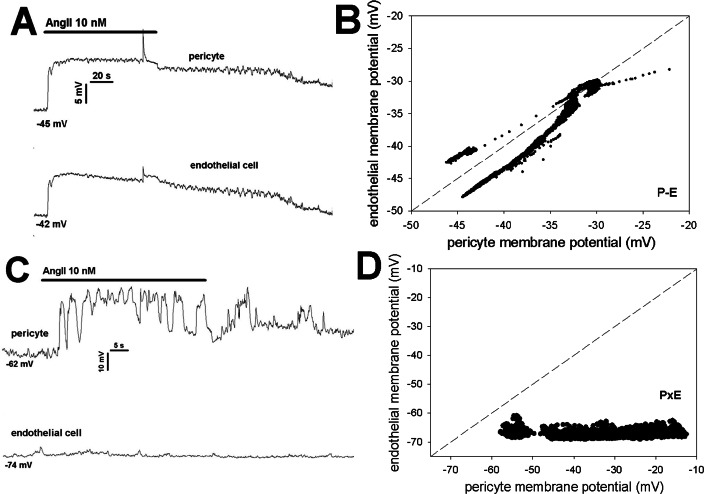
Simultaneous pericyte, endothelial membrane potential responses to AngII with and without cell contact. **A.** Example of AngII induced membrane potential responses of a pericyte and an endothelial cell physically associated with one another along the DVR wall (as in Fig 5A). Similar time dependent variations persisted throughout the record. Resting potentials prior to AngII were -45 mV and -42 mV. **B.** Individual data points of the record in panel A are shown superimposed upon the line of identity. Throughout most of the record, the endothelium remained hyperpolarized relative to the pericyte. **C.** Example of AngII induced membrane potential responses of a pericyte and an endothelial cell when cells were dissociated (as in Fig 5B). Membrane potential variations were observed in the pericyte but not in the endothelial cell. Resting potentials prior to AngII were -62 mV and -74 mV. **B.** Individual data points of panel A are shown superimposed upon the line of identity. Pericyte response occurred but a concomitant endothelial response was absent.

**Fig 9:**
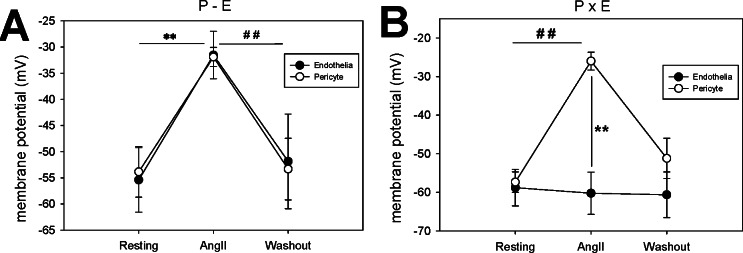
Summary of simultaneous pericyte and endothelial membrane potential responses to AngII with and without cell contact. **A.** Summary of average membrane potential before (resting), during peak depolarization (AngII), and after washout (n = 6, P-E configuration, as in Fig 5A). The depolarizations (mean ± SEM) were significant (repeated measures ANOVA, DF = 17, F = 12.9, P < 0.01). By Holm-Sidak multiple comparison; **, P < 0.01 resting vs AngII, ##, P < 0.01 AngII vs washout. Pericyte and endothelial depolarizations were nearly identical (no significant differences between cell types). **B.** Summary of average membrane potentials before, during AngII, and after washout (n = 6, PxE configuration as in Fig 5A). The pericyte depolarizations (mean ± SEM) were significant (repeated measures ANOVA, DF = 17, F = 53.5, P < 0.01). By Holm-Sidak multiple comparison; ##, P < 0.01 resting vs AngII or AngII vs washout. In contrast to the P-E configuration, in the absence of pericyte contact, AngII did not alter endothelial membrane potential; (group t-test, ** P < 0.01 endothelia vs pericytes during AngII exposure).

In another series, we measured simultaneous membrane potential responses of pericytes and endothelia to AngII exposure when the cells were either in communication on an intact vessel as in **Fig 5A** (P-E), or isolated from one another as in **Fig 5B** (PxE). As previously reported [18], when in contact, resting membrane potentials were very similar, with endothelia slightly hyperpolarized relative to pericytes (**Fig 7A and 7B**). When pericytes were fully isolated from the endothelium, resting potentials remained similar in magnitude (**Fig 7C**) but were not correlated (**Fig 7D**) and the relative endothelial hyperpolarization no longer persisted (compare **Fig 7A and 7C**). Finally, when AngII response was measured in partially denuded vessels as in **Fig 5A** (P-E), pericyte and endothelial membrane potentials remained highly coordinated (**Fig 8A and 8B**) [18]. In contrast, total isolation of pericytes from endothelia as in **Fig 5B** (PxE) completely eliminated AngII induced endothelial depolarizations (**Fig 8C and 8D**). As summarized in **Fig 9** these results were highly consistent.

## Discussion

Syncytial communication of the vasculature is thought to be most prominent in microvessels [9, 19, 20]. The ability of the vessel wall to accommodate mural conduction of depolarizing and hyperpolarizing signals probably serves to extend their spatial influence to upstream locations [19, 21]. In the renal medulla, where access, in vivo is impossible, this is an unproven but inviting prospect. It is widely believed that countercurrent trapping leads to progressive hypoxia with medullary depth, predisposing to tissue injury [22, 23]. Hypothetically, the ability of the DVR wall to carry vasodilatory hyperpolarization toward their parent juxtamedullary efferent arterioles could be important to matching metabolic demands and blood supply. By that scheme, hyperpolarization from adenosine, NO, CO, EETs or other diffusible activators released from interbundle epithelia might generate feedback responses to rescue their oxygenation [24–26]. DVR also generate their own intrinsic signals in the form of endothelium dependent relaxing and hyperpolarizing factors. Intrinsic NO release has major influence on the tone of isolated DVR [19]. Moreover, strong inward rectifier K^+^ channels and K_ATP_ channels are expressed to favor their combined syncytial hyperpolarization by extracellular K^+^ ion or other EDHFs [27, 28]. Finally, not to be discounted in this scheme, mechanical forces such as endothelial shear or tissue compression via ureteral peristalsis probably stimulate electrical responses within the DVR wall. Shear dependent NO release has been demonstrated [29] and mechanical perturbation of the abluminal surface induces brisk, reversible depolarizations and calcium elevations that spread along the vessel axis by mural conduction. The latter were found to be sensitive to either gap junction or L-type VGCC blockade [30].

Similar to juxtamedullary efferent arteriolar smooth muscle [31], DVR express VGCC and electrophysiological recordings have shown nifedipine-sensitive L-type Ca^2+^ / Ba^2+^ currents in DVR pericytes [4]. Stimulation by vasoconstrictors induces depolarization that opens VGCC following activation of CaCC. Concomitant suppression of K^+^ conductance augments durability of the attendant rise in membrane potential [6, 7]. As such, control of membrane potential and conduction of it between cells regulates DVR vessel tone. Given that DVR supply the medulla at various depths, depending on their location within vascular bundles, it is reasonable to surmise that similar events may play a pivotal role to regulate blood flow distribution within the medulla. This notion is favored by studies of changes in medullary blood following global VGCC blockade [32–34].

The goal of the present study was to define the routes by which membrane potential can be conducted within the DVR wall. Depolarizing responses might spread via gap junctions between pericytes. Alternately, the conduction might involve only myo-endothelial conduction such that membrane potential changes originating from a pericyte must conduct in sequence through the endothelium and then to nearby pericytes. Finally, the origins of endothelial voltage responses to agonists are uncertain. Do AngII responses of endothelia depend upon endothelial receptors, or are the responses conducted from temporal changes that occur within adjacent pericytes? Since gap junction blockers have nonspecific actions and genetic manipulation of connexins might alter compositions of connexon proteins, we utilized physical disruption of cell-to-cell communication to explore these questions. Our results show that pericytes on the wall of an isolated DVR segment have nearly identical membrane potentials (**Fig 1**). Within the limits of spatial separation afforded by DVR isolation (i.e., 300 to 1000 microns), the syncytial sharing of voltage between pericytes seems robust.

Connexons form gap junction intercellular channels with variable conductance, open probabilities and sub conductance states [9, 35]. As such, they might be regulated by differences in membrane potential between the cells they couple, or by covalent modifications of their subunits downstream of signaling events [36]. For example phosphorylation of connexins 37, 40 and 43 affect cell-to-cell communications in varying ways [36–38]. We measured the equivalence of pericyte membrane potentials after vasoactive stimulation by AngII, AVP or ET1, each of which are known to constrict DVR. A persistent, robust coordination between cells was always retained (**Fig 2**). Moreover, even after pericytes were isolated from the endothelium, they coordinated responses between themselves, implying that gap junctions between pericytes are sufficient to conduct electrical activity (**Fig 4**).

We previously demonstrated that endothelial membrane potential closely tracks that of nearby pericytes, while remaining a few millivolts hyperpolarized to them. Likely, this relationship provides a braking effect so that pericyte depolarization is blunted and VGCC mediated Ca^2+^ entry and consequent DVR constriction is self-limited. In contrast to the close parallel voltage changes of adjacent pericytes and endothelium, parallel changes of cytoplasmic Ca^2+^ (Ca_CYT_) does not occur. When AngII induces oscillatory or persistent increases in pericyte Ca_CYT_, endothelial Ca_CYT_ is suppressed [39, 40]. Similarly, mechanical stimulation of the pericyte surface depolarizes them, elevates pericyte Ca_CYT_ and generates conducted Ca_CYT_ elevations along the vessel axis. During that response, concomitant endothelial Ca_CYT_ changes are small or absent [30]. Thus, any monovalent ion movements responsible for coordination of membrane potential fluctuations may not be accompanied by cell-to-cell divalent ion fluxes. If divalent flux across gap junctions does occur the effects on cytoplasmic concentrations may be rapidly blunted cytoplasmic buffering mechanisms [41].

The existence of AngII receptors on DVR endothelia has been uncertain [39]. It is possible that endothelial responses are mediated by the coordination of membrane potential changes alone (**Figs 5–8**), or by diffusion of secondary signaling molecules across myo-endothelial junctions. We separated the cells of native isolated DVR to test the possibilities (**Fig 5**). Square wave pericyte depolarizations imposed by voltage clamp were conducted to endothelia and sensitive to chemical gap junction blockade (**Fig 6**). Mechanical removal of pericytes from the DVR wall eliminated close correlation between pericyte and endothelial resting potentials (**Fig 7**). Moreover, it fully eliminated the ability of AngII to depolarize DVR endothelia (**Figs 8 and 9**). These data support the interpretation that expression of AngII receptors on DVR endothelia might account for DVR endothelial electrical responses. Rather, conductance via myo-endothelial junctions may yield such changes. AngII lowers Ca_CYT_ in DVR endothelia [39, 40]. This too might result from depolarization. If endothelial VGCC are absent and extracellular calcium is conducted into the endothelial cytoplasm through voltage independent cation channels, e.g., TRP isoforms [42], the reduction in electrochemical gradient that accompanies depolarization might be sufficient to diminish the Ca^2+^ influx rate and account for Ca_CYT_ suppression. The precise relationship between endothelial Ca_CYT_ and membrane potential remains an area of investigation [43].

Connexin (Cx) isoforms associate in homologous and heterologous combinations to form gap junction proteins that dock to form channel-like conduits between cells [44]. Immunostaining of the DVR wall identified expression of Cx37, Cx40 and Cx43 which are commonly found in the vasculature [45]. Those isoforms and Cx45 [46] are expressed in arterioles in the renal cortex where vascular gap communication may coordinate oscillatory pressure fluctuations of adjacent nephrons [47, 48], modify transmission of systemic pressure to glomeruli and play roles in renin release [49–51]. Such behavior is not unique to the kidney. Recognition of the existence of vasomotion and calcium waves in the microvasculature of various organ beds has been longstanding. It is broadly accepted that oscillatory variations of membrane potential and Ca_CYT_, manifested as vasomotion, is coordinated via smooth muscle and endothelial gap junctions [52]. Disease states such as atherosclerosis and diabetes lead to changes in vasoactivity, but the physiological role(s) and consequences of modifying normal activity continues to be unclear. Studies in the renal cortex where surface vessels are accessible have yielded some intriguing results. The range over which such coupling occurs had been thought to be small. Simultaneous measurements of nephron activity using micropuncture methods are technically limited, but more recent observations over broader areas of the cortex using laser-speckle imaging has revealed extended synchronization. This implies possible coordination via interlobular arterioles and their attendant regions of perfusion [53, 54]. Study of the implications of this seminal finding for salt and water homeostasis, glomerular hypertension, and kidney disease is in infant stages and will be of much interest. Diminished synchronization between nephrons in hypertensive states has already been shown [55]. With regard to the renal outer medulla, where vascular bundles are inaccessible in vivo, studies of coordination of tissue perfusion remain absent.

There are limitations to interpretation of these studies. Outer medullary DVR are inaccessible in vivo in a region of the kidney where oxygen tensions are low and osmolality higher than systemic plasma. The precise concentrations of electrolytes and urea cannot be known with certainty. Our experimental conditions cannot replicate that environmental milieu its osmolality or oxygen tensions. Our buffers do not contain the repertoire of paracrine agents that must certainly exist in renal outer medullary vascular bundles, in vivo. As such, extrapolation of these results to predict renal medullary physiology must be done with uncertainty and caution.
